# Can an electronic ICU support timely renal replacement therapy in resource-limited areas of the developing world

**DOI:** 10.1186/cc14584

**Published:** 2015-03-16

**Authors:** S Gupta, A Kaushal, S Dewan, A Varma

**Affiliations:** 1 Fortis Escorts Heart Institute, New Delhi, India; 2 Fortis Memorial Research Institute, Gurgaon, India

## Introduction

Timely availability of a kidney specialist poses a formidable challenge in ICUs located in tier II and tier III cities of the developing world. Renal replacement therapy (RRT) is often required in the ICU for acute renal failure patients but availability of a nephrologist/ specialist is scarce, leading to unnecessary and risky transfer to higher centers in metropolitans or even worse to death. We explored whether a remotely monitored ICU - an electronic ICU (eICU) - would help mitigate this demand-supply gap.

## Methods

This retrospective study was conducted at four Critinext affiliates where the eICU was being used to provide 24 × 7 support on 89 ICU beds from a remote command center with intensivist and other requisite staff. The eICU had complete access to the patient's real-time vitals, hemodynamic parameters, imaging, laboratory values, audiovisuals and appropriately engineered smart alerts. The eICU model was further extended in initiating and getting RRT done in patients whenever deemed necessary in times of unavailability of a specialist at the same site. Patient baseline demographics, including risk factors, severity score, all-cause mortality at 30 days, transfers to higher center for RRT and its prevention were recorded. Descriptive analysis was performed. Between-group comparison was performed by applying the chi-squared statistic, significance was assumed at a value of *P *< 0.05. Out of a total of 5,146 admissions, 752 inpatient files with acute kidney injury/acute renal failure were reviewed, January to July 2013 (*n = *373) and July 2013 to January 2014 (*n = *379) pre and post eICU implementation respectively.

## Results

While baseline demographics and the patient profile in the two groups did not show statistically significant difference, mean APACHE II score was 14.25 ± 1.94 and 14.65 ± 1.76 pre and post eICU respectively; there was a statistically significant difference in all-cause mortality at 30 days which decreased from 31 (8.3%) to 16 (4.2%) pre and post eICU respectively, a reduction of >49% (*P *= 0.030) and transfer out for RRT came down by >77%, from 15 (4%) to two (0.5%) post eICU implementation (*P *< 0.002).

## Conclusion

Over the years there is now broad consensus over the benefits of eICU intervention in deprived areas [1]. There is now a need for a paradigm shift to elevate specialized care to improve outcomes. Our small study has clearly indicated the benefits in outcome and economics even while providing intervention in such remote areas. An eICU as a bridge to the demand-supply gap needs to be explored and utilized further to its full potential in the emerging world.
